# Genomic expression and single-nucleotide polymorphism profiling discriminates chromophobe renal cell carcinoma and oncocytoma

**DOI:** 10.1186/1471-2407-10-196

**Published:** 2010-05-12

**Authors:** Min-Han Tan, Chin Fong Wong, Hwei Ling Tan, Ximing J Yang, Jonathon Ditlev, Daisuke Matsuda, Sok Kean Khoo, Jun Sugimura, Tomoaki Fujioka, Kyle A Furge, Eric Kort, Sophie Giraud, Sophie Ferlicot, Philippe Vielh, Delphine Amsellem-Ouazana, Bernard Debré, Thierry Flam, Nicolas Thiounn, Marc Zerbib, Gérard Benoît, Stéphane Droupy, Vincent Molinié, Annick Vieillefond, Puay Hoon Tan, Stéphane Richard, Bin Tean Teh

**Affiliations:** 1 Laboratory of Cancer Genetics, Van Andel Research Institute, 333 Bostwick Avenue NE, Grand Rapids, MI, 49503, USA; 2 NCCS-VARI Translational Cancer Research Laboratory, National Cancer Centre Singapore, 11 Hospital Drive, 169610 Singapore; 3 Department of Medical Oncology, National Cancer Centre Singapore, 11 Hospital Drive, 169610 Singapore; 4 Department of Epidemiology and Public Health, National University of Singapore, 16 Medical Drive, 117597 Singapore; 5 Department of Pathology, Singapore General Hospital, Outram Road, 169608 Singapore; 6 Northwestern University Feinberg School of Medicine, 251 E. Huron, Chicago, IL, 60611, USA; 7 Department of Urology, Iwate Medical University School of Medicine, 19-1, Uchimaru, Marioka, 020-8505 Japan; 8 Laboratory of Computational Biology, Van Andel Research Institute, 333 Bostwick Avenue NE, Grand Rapids, MI, 49503, USA; 9 Laboratory of Molecular Epidemiology, Van Andel Research Institute, 333 Bostwick Avenue NE, Grand Rapids, MI, 49503, USA; 10 Laboratoire de Génétique, Hôpital Herriot, Batiment 7, Place d'Arsonval, 69437 Lyon Cedex 03, France; 11 Laboratoire d’Anatomie Pathologique, Hôpital de Bicêtre, AP-HP, 78 rue du Général Leclerc, 94275 Le Kremlin-Bicêtre Cedex, France; 12 Department of Biology and Pathology, Institut de Cancérologie Gustave Roussy, 39 rue Camile Desmoulins, 94805 Villejuif, France; 13 Department of Urology, Hôpital Cochin, 27 rue du Faubourg Saint-Jacques, 75679 Paris Cedex 14, France; 14 Service d'Urologie, Hôpital Necker, AP-HP, 149, rue de Sevres, 75743 Paris, France; 15 Service d’Urologie, Hôpital de Bicêtre, AP-HP, 78 rue du Général Leclerc, 94275 Le Kremlin-Bicêtre, France; 16 Service d'Anatomie Pathologique, Hôpital Saint Joseph, 185 rue Raymond Losserand, 75674 Paris Cedex, France; 17 Laboratoire d'Anatomie Pathologique, Hôpital Cochin, 27 rue du Faubourg Saint-Jacques, 75679 Paris Cedex 14, France; 18 Consultation d'Oncogénétique Spécialisée, Service d'Urologie, Hôpital de Bicêtre, 78 rue du General Leclerc, 94275 Le Kremlin-Bicêtre, France; 19 Génétique Oncologique EPHE-INSERM U753 and Faculté de Médecine Paris-Sud, Le Kremlin-Bicêtre, and Institut de Cancérologie Gustave Roussy, 39 rue Camille Desmoulins, 94805 Villejuif, France

## Abstract

**Background:**

Chromophobe renal cell carcinoma (chRCC) and renal oncocytoma are two distinct but closely related entities with strong morphologic and genetic similarities. While chRCC is a malignant tumor, oncocytoma is usually regarded as a benign entity. The overlapping characteristics are best explained by a common cellular origin, and the biologic differences between chRCC and oncocytoma are therefore of considerable interest in terms of carcinogenesis, diagnosis and clinical management. Previous studies have been relatively limited in terms of examining the differences between oncocytoma and chromophobe RCC.

**Methods:**

Gene expression profiling using the Affymetrix HGU133Plus2 platform was applied on chRCC (n = 15) and oncocytoma specimens (n = 15). Supervised analysis was applied to identify a discriminatory gene signature, as well as differentially expressed genes. High throughput single-nucleotide polymorphism (SNP) genotyping was performed on independent samples (n = 14) using Affymetrix GeneChip Mapping 100 K arrays to assess correlation between expression and gene copy number. Immunohistochemical validation was performed in an independent set of tumors.

**Results:**

A novel 14 probe-set signature was developed to classify the tumors internally with 93% accuracy, and this was successfully validated on an external data-set with 94% accuracy. Pathway analysis highlighted clinically relevant dysregulated pathways of c-erbB2 and mammalian target of rapamycin (mTOR) signaling in chRCC, but no significant differences in p-AKT or extracellular HER2 expression was identified on immunohistochemistry. Loss of chromosome 1p, reflected in both cytogenetic and expression analysis, is common to both entities, implying this may be an early event in histogenesis. Multiple regional areas of cytogenetic alterations and corresponding expression biases differentiating the two entities were identified. Parafibromin, aquaporin 6, and synaptogyrin 3 were novel immunohistochemical markers effectively discriminating the two pathologic entities.

**Conclusions:**

Gene expression profiles, high-throughput SNP genotyping, and pathway analysis effectively distinguish chRCC from oncocytoma. We have generated a novel transcript predictor that is able to discriminate between the two entities accurately, and which has been validated both in an internal and an independent data-set, implying generalizability. A cytogenetic alteration, loss of chromosome 1p, common to renal oncocytoma and chRCC has been identified, providing the opportunities for identifying novel tumor suppressor genes and we have identified a series of immunohistochemical markers that are clinically useful in discriminating chRCC and oncocytoma.

## Background

Epithelial renal cell carcinoma (RCC) is the most common malignancy of the adult kidney. RCC is a clinicopathologically heterogeneous disease that is traditionally classified by morphology into clear cell, papillary, chromophobe, and collecting duct carcinoma. Chromophobe renal cell carcinoma (chRCC) and renal oncocytoma are two distinct but related entities, with strong morphologic and genetic similarities [1]. Distinguishing between the two tumors may present a significant diagnostic challenge on routine hematoxylin-eosin stained sections, especially in cases with features resembling both chRCC and oncocytoma, oncocytoma with associated invasion and even metastasis [2], and the eosinophilic variant of chRCC.

ChRCCs account for about 4-8% of all renal tumors, with a more favorable prognosis relative to clear cell renal cell carcinoma, which comprises the majority of all RCCs [3]. On the other hand, oncocytoma is the most common benign renal tumor, comprising 5-8% of resected renal masses. The overlapping characteristics of these entities may be explained by a possible common origin from the intercalated cells of the distal tubule [4]. Patients with Birt-Hogg-Dubé syndrome, a familial multi-tumor syndrome linked to mutation of the *BHD *gene, exhibit bilateral oncocytomas, chRCC and hybrid tumors [5,6].

In our previous gene expression profiling studies of a limited number of chRCC and oncocytoma [7], we demonstrated that both tumors showed strong similarities in expression patterns suggesting a common underlying biology [8] and this was supported by subsequent expression profiling studies by other groups [9]. We hypothesized that more effective discrimination might be achieved with a larger sample number with additional analyses, and that the differences might shed light on the underlying genetic drivers of tumorigenesis, diagnosis and clinical management. We set out to perform a comprehensive characterization of both entities by integrating gene expression and high resolution single-nucleotide polymorphism (SNP) profiling, proceeding to identify a useful and valid molecular predictor, as well as identifying novel immunohistochemical markers for each entity.

## Methods

### Gene expression profiles

A total of 30 frozen primary kidney tumors (15 chRCC and 15 oncocytomas) were obtained from the French Kidney Tumors Consortium, University of Chicago, Northwestern University, and Spectrum Health Hospital (Grand Rapids, MI). Each sample was confirmed by pathologic analysis and anonymized prior to the study. A portion of the tumor sample was frozen in liquid nitrogen immediately after surgery and stored at -80°C. Total RNA was isolated from the frozen tissues using Trizol reagent (Invitrogen, Carlsbad, CA) and purified using the RNEasy kit (Qiagen). Gene expression profiling was performed as previously described using the HGU133 Plus 2.0 Affymetrix GeneChip platform, with 54,675 distinct transcripts assayed [10]. An external GEO data-set of gene expression profiles of oncocytomas and chRCC from Cornell University was obtained for validation (GSE12090) [11]. Data for this study has been uploaded publicly in the Gene Expression Omnibus, with the accession number GSE19982.

### DNA single-nucleotide polymorphism (SNP) arrays

DNA from an independent set of 6 chRCC and 8 oncocytomas obtained from the Cooperative Human Tissue Network were isolated using a Jetquick DNA extraction kit (Genomed, Lohne, Germany) according to the manufacturer's protocol. The SNP assay was performed according to the manufacturer's instructions using the Affymetrix GeneChip Mapping 100 K array (Affymetrix, Santa Clara, CA). The raw SNP array data was processed by Affymetrix GeneChip Genotyping analysis (GTYPE v.3) and human genome reference of NCBI build 36 was used for analysis.

### Statistical analyses for expression data

Statistical analyses were performed in the statistical environment R 2.6.0, utilizing packages from the Bioconductor project [12]. The robust multichip average (RMA) algorithm was used to perform pre-processing of the CEL files, including background adjustment, quartile normalization and summarization. For purposes of hierarchical analysis using complete linkage analysis, probe set filtering for coefficient of variation (≥0.05, with at least 2 samples showing log_2 _value expression of 8) was performed. Significance analysis of microarrays (SAM) on unfiltered data based on two-class unpaired analysis, assumption of unequal group variances and 10,000 permutations was used to derive a list of probe sets differentially expressed between tumor subclasses, and ordered by relative fold-change [13]. A maximum false discovery rate threshold was defined as 0.05.

For derivation of a small gene classifier, we used prediction analysis of microarrays (PAM), an R implementation of nearest shrunken centroids methodology with 10-fold cross validation over 100 gene thresholds and an offset percentage of 30% on unfiltered data [14]. A maximum acceptable cross-validated misclassification error was defined as ≤ 10%. The smallest predictor corresponding to this cross-validated error was selected for external validation. We inferred cytogenetic profiles for the tumors through the use of a refinement of the comparative genomic microarray analysis (CGMA) algorithm [15], which predicts chromosomal alterations based on regional changes in expression. Briefly, relative expression profiles *R *were generated from the single channel tumor expression profiles (*T*) and the mean expression values of 12 single channel cortical kidney expression profiles (*N*) such that *R *= log_2_(*T*) - log_2_(*N*).

### Pathway analysis

KEGG pathway and gene ontology (GO) analysis of enriched gene sets was performed using hypergeometric tests available in the GOstats package in Bioconductor after having identified unique genes with corresponding annotations. For KEGG pathway analysis, the p-value threshold was 0.01. For GO analysis, conditional testing was performed, and the threshold for p was 0.001. Molecular function, biologic process, and cellular component analyses were performed.

### DNA copy number analysis

DNA copy number (CN) was calculated based on the allele intensity of each SNP probe on the array using dChip [16]http://biosun1.harvard.edu/complab/dchip/. Information about the cytobands and the physical position of all SNPs was obtained from Affymetrix and UCSC genome bioinformatics database (NCBI Build 36.1) http://genome.ucsc.edu. The working criteria for loss or gain are defined as the chromosomal region with at least four consecutive SNPs with CN < 1.6, or at least four consecutive SNPs with CN > 3.5, respectively. Copy number alteration (CNA) regions were identified when more than 30% of the samples showed copy number loss or gain.

### **Immunohistochemistry**

Immunohistochemical staining was performed on an independent set of chRCC (n = 11) and oncocytomas (n = 7). Aquaporin 6 and synaptogrin 3 were selected from the PAM (Table 1). Parafibromin (218578_at) (2-fold expression difference) and cytokeratin 7 (209016_s_at) were selected from the SAM analysis of the gene expression profiles for validation. Candidate marker choice was determined by factors including fold-change, specificity, biological and clinical interest. CK7 was selected as a marker to ascertain the additional benefit of routine pathologic practice in the samples. Briefly, following blocking and antigen retrieval, 4-micron sections on coated slides were incubated with the following antibodies: a mouse anti-cytokeratin 7 monoclonal antibody (DakoCytomation, Carpinteria, CA, 1:50, cytoplasmic staining), a polyclonal rabbit anti-human aquaporin 6 (AQP6, Alpha Diagnostic International, San Antonio, TX, 1:100, overnight at 4°C, membranous staining), polyclonal goat anti-synaptogyrin 3 N-18 and C-18 antibodies (SYNGR3, Santa Cruz Biotechnology, Santa Cruz, CA, cytoplasmic and membranous staining), a mouse monoclonal antibody specific for parafibromin (1:250, 1 hour at room temperature, nuclear staining) [17], a rabbit monoclonal anti-HER2 antibody (Neomarker RM 9103-S clone SP3, 1:200, membranous staining), a phospho-AKT (Ser473) antibody (Cell Signaling Technology, 1:30, cytoplasmic and nuclear staining). For the latter two antibodies, 22 chromophobe RCC and 8 oncocytoma specimens were available. For p-AKT, staining in the stromal and the tumor cell compartments was separately assessed. Subsequent reactions were performed with biotin-free HRP enzyme labeled polymer of EnVision Plus detection system (DakoCytomation). All slides were examined by a pathologist in a blinded fashion.

**Table 1:** Predictor derived via nearest shrunken centroid method for sample classification of chromophobe RCC and oncocytoma.

Affymetrix Probe ID	Gene description	ChRCC-score*	Oncocytoma-score*	Fold change**
216219_at	aquaporin 6	-0.1972	0.1972	0.20
240304_s_at	transmembrane channel-like 5	0.1247	-0.1247	13.0
208435_s_at	aquaporin 6	-0.1218	0.1218	0.22
205691_at	synaptogyrin 3	0.1108	-0.1108	3.75
230110_at	mucolipin 2	-0.0731	0.0731	0.15
52940_at	single immunoglobulin and toll-interleukin receptor (TIR) domain	0.0577	-0.0577	3.28
217879_at	cell division cycle 27 homolog (S. cerevisiae)	-0.0403	0.0403	0.
222574_s_at	DEAH (Asp-Glu-Ala-His) box polypeptide 40	-0.0228	0.0228	0.48
218921_at	single immunoglobulin and toll-interleukin receptor (TIR) domain	0.0205	-0.0205	3.16
1557137_at	transmembrane protein 17	-0.0178	0.0178	0.40
230644_at	leucine rich repeat and fibronectin type III domain containing 5	0.0172	-0.0172	4.90
223087_at	enoyl Coenzyme A hydratase domain containing 1	-0.0167	0.0167	0.42
203039_s_at	NADH dehydrogenase (ubiquinone) Fe-S protein 1	-0.0093	0.0093	0.46
202502_at	acyl-Coenzyme A dehydrogenase	-0.0089	0.0089	0.35

* A class discriminant score derived from nearest shrunken centroids methodology.

** Fold change of gene expression in chRCC relative to oncocytoma

## Results

### Gene expression profiling

**Figure 1:**
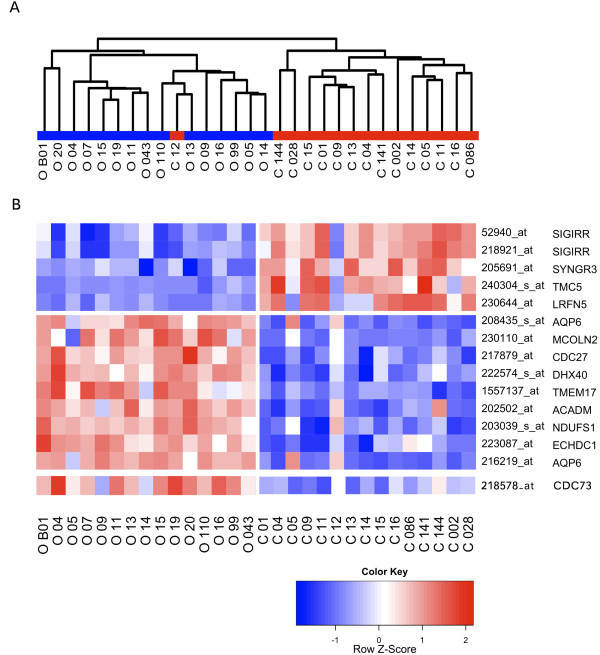
**Discrimination of oncocytoma and chromophobe RCC by expression profiling**. (A) A dendrogram showing an unsupervised hierarchical cluster of the filtered data showing clustering of oncocytoma and chromophobe RCC. The color bar here separates oncocytoma (O) from chromophobe RCC (C). (B) A heatmap of the predictor genes. Red denotes relative overexpression and blue denotes relative under-expression. Relative parafibromin (CDC73) expression is also reported here in chromophobe RCC and oncocytoma, distinct from the 14-transcript predictor.

We visualized the 30 expression profiles of chRCC and oncocytoma by hierarchical clustering upon a filtered data-set of 8,995 transcripts. Clear partitioning of the two entities into separate classes was observed (Figure 1). PAM yielded excellent cross-validated discrimination over a series of thresholds [Additional file 1: Figure S1]. A gene predictor comprising 14 probe sets was identified (Table 1), which yielded an overall accuracy of 93% in the internal data-set (28/30) (Table 2). The same predictor successfully classified 17 of 18 samples in the external dataset from Cornell University, corresponding to an overall accuracy of 94% (Table 2). 5,210 probe sets were found to be differentially expressed between the two entities as identified using SAM at a delta of 1.4, with a false discovery rate of 0.03 corresponding to an estimated 222 probe sets [Additional file 2: Table S1]. 2,564 number of probe sets were relatively overexpressed in chRCC, and 2,646 transcripts relatively underexpressed in chRCC.

**Table 2:** Predictor performance in sample classification of internal and external data-sets.

		Gene predictor (14 probe sets)	
		Predicted chRCC	Predicted oncocytoma
Internal Data-Set	chRCC	13/15 (87%)	2/15 (13%)
	Oncocytoma	0/15 (0%)	15/15 (100%)

External Data-Set	chRCC	8/9 (89%)	1/9 (11%)
(Cornell)	Oncocytoma	0/9 (0%)	9/9 (100%)

### DNA copy number profiling and comparative genomic microarray analysis

**Figure 2:**
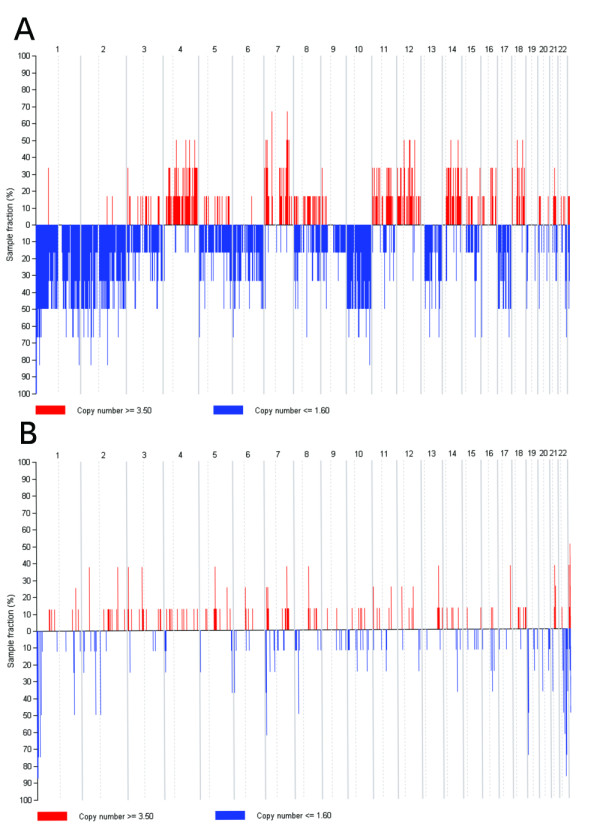
**High throughput SNP analysis data in chRCC (above) and oncocytoma (below) showing multiple chromosomal copy number alterations in chRCC, but not in oncocytoma**.

**Figure 3:**
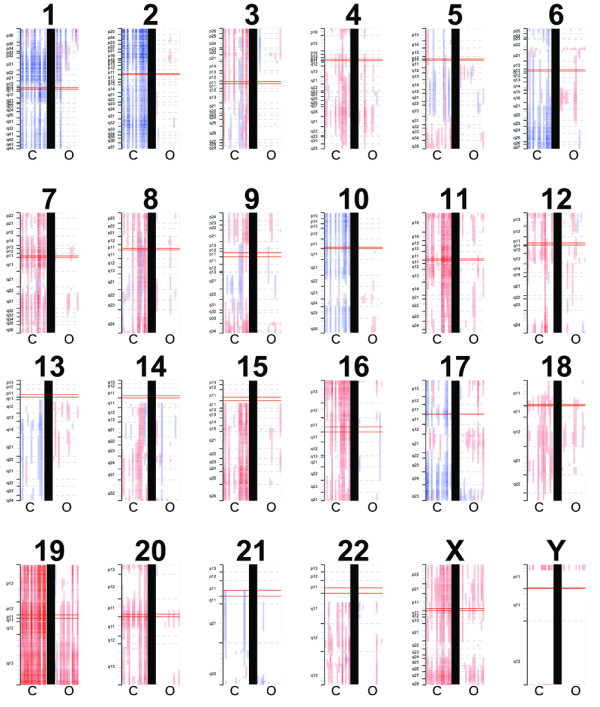
**Chromosomal ideograms derived from comparative genomic microarray analysis from expression profiles showing tumor regional expression biases**. For each ideogram, a tumor is represented by an individual vertical bar with chRCC (C) on the left and oncocytoma (O) on the right. Red denotes an increase in predicted copy number, and blue a decrease.

We report copy number gains that were detected in chromosomes 4, 7, 11, 12, 14q, and 18q (Figure 2). Whole chromosomal losses of chromosome 1, 2, 6, 10, 13, 17, and 21 represent a unique copy number loss profile for chRCC. For renal oncocytoma, losses of chromosome 1p were noted. A CGMA (Figure 3) derived from the expression data yielded regional expression biases consistent with that reported by DNA copy number profiling. We report in particular that our high throughput methods demonstrate that there is a common gene alteration to both tumors (loss of chromosome 1p), which may represent an early event common in the histogenesis of both tumors. In particular, in oncocytoma, this loss appears restricted to the terminal end of 1p.

### Pathway Analysis

Pathway and GO analysis was performed on the SAM analysis, demonstrating an enrichment of genes involved in metabolic pathways in oncocytomas relative to chRCC (Table 3). These metabolic pathways include oxidative phosphorylation, amino acid metabolism, and fatty acid metabolism. Conversely, high expression of genes involved in cell adhesion, immune receptor signaling as well as proliferative pathways such as c-erbB2 (Her-2/neu) and mammalian target of rapamycin (mTOR) signaling are detected in chRCC. GO analyses performed supported these results [Additional file 3: Table S2], highlighting that mitochondrial genes were highly overrepresented among genes relatively overexpressed in oncocytomas, whereas tight junction genes were similarly overrepresented among genes overexpressed in chRCC.

**Table 3:** Molecular pathways discriminating chRCC and oncocytoma.

Pathways relatively upregulated in oncocytoma						
KEGGID	Pvalue	OddsRatio	ExpCount	Count	Size	Term
280	0	6.206	4	17	44	Valine, leucine and isoleucine degradation
640	0	6.349	3	13	33	Propanoate metabolism
190	0	3.093	11	27	114	Oxidative phosphorylation
970	0	4.488	4	12	38	Aminoacyl-tRNA biosynthesis
20	0.001	4.833	3	9	27	Citrate cycle (TCA cycle)
330	0.001	4.032	3	10	34	Arginine and proline metabolism
4120	0.003	3.13	4	11	45	Ubiquitin mediated proteolysis

**Pathways relatively upregulated in chRCC**						
**KEGGID**	**Pvalue**	**OddsRatio**	**ExpCount**	**Count**	**Size**	**Term**

4660	0	3.263	9	23	93	T cell receptor signaling pathway
4662	0	3.945	6	18	63	B cell receptor signaling pathway
4514	0	2.505	12	26	129	Cell adhesion molecules (CAMs)
4670	0	2.676	10	23	108	Leukocyte transendothelial migration
5220	0	3.05	7	18	76	Chronic myeloid leukemia
5212	0	2.977	7	17	73	Pancreatic cancer
4520	0.001	2.823	7	17	76	Adherens junction
5130	0.001	3.335	5	13	51	Pathogenic Escherichia coli infection - EHEC
5131	0.001	3.335	5	13	51	Pathogenic Escherichia coli infection - EPEC
4530	0.001	2.277	11	22	117	Tight junction
4664	0.001	2.65	7	16	75	Fc epsilon RI signaling pathway
4620	0.003	2.268	10	19	101	Toll-like receptor signaling pathway
4012	0.003	2.372	8	17	87	ErbB signaling pathway
564	0.003	2.621	6	14	66	Glycerophospholipid metabolism
4150	0.004	2.964	4	11	47	mTOR signaling pathway
5120	0.004	2.523	6	14	68	Epithelial cell signaling in Helicobacter pylori infection
4210	0.005	2.293	8	16	84	Apoptosis
4070	0.006	2.317	7	15	78	Phosphatidylinositol signaling system
4540	0.007	2.124	9	17	95	Gap junction
4912	0.009	2.07	9	17	97	GnRH signaling pathway
5221	0.01	2.536	5	11	53	Acute myeloid leukemia

### **Immunohistochemical findings**

**Figure 4:**
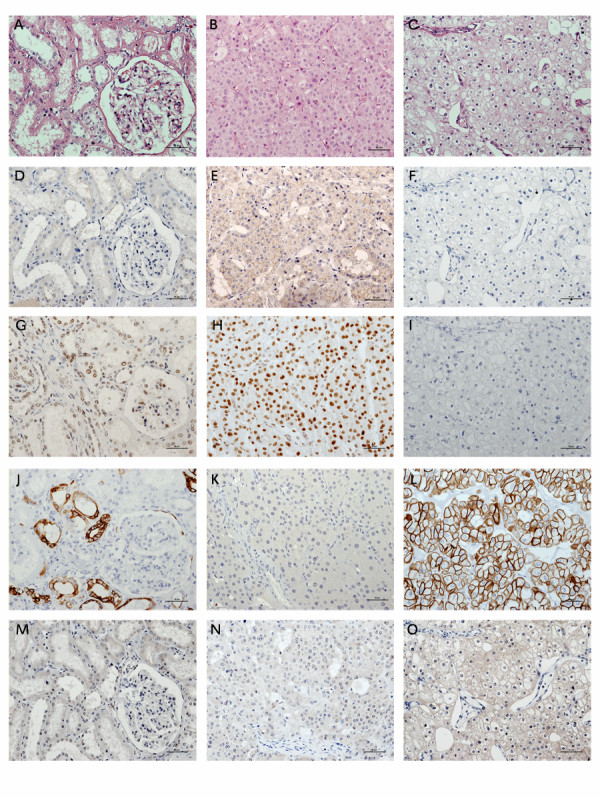
**Immunohistochemical profiling of renal oncocytoma and chromophobe RCC**. (A) - (C) Hematoxylin and eosin stains of normal cortical kidney tissue, oncocytoma and chromophobe RCC respectively; (D) - (F) Aquaporin 6 immunostaining showing membranous staining in oncocytoma but absent staining in chromophobe RCC; (G) - (I) Parafibromin immunostaining showing strong nuclear expression in oncocytoma and tubular epithelium but absent staining in chromophobe RCC; (J) - (L) Cytokeratin 7 immunostaining showing distinct cytoplasmic staining in chromophobe RCC but absent staining in oncocytoma; (M) - (O) Synaptogyrin 3 immunostaining showing cytoplasmic staining in chromophobe RCC but absent staining in oncocytoma.

The immunohistochemical profiling is summarized in Table 4 and Figure 4, the results of which were consistent with the mRNA quantitation by microarrays. The immunoreactivity of chRCC to cytokeratin 7 was higher than that of oncocytomas and normal kidney. For parafibromin, clear differential staining was noted, with predominantly nuclear expression in oncocytomas, and absent expression in chRCC. For synaptogyrin-3, both N-18 and C-18 antibodies yielded a similar signal, but the N-18 antibody yielded a crisper result though the maximal signal was distinctly weaker compared to AQP6, for which crisp membranous staining was noted in oncocytoma, but not in chRCC. For p-AKT, there was an apparent, but non-significant higher immunoreactivity in chRCC than oncocytoma, particularly in the stromal cells relative to the tumor cells. For extracellular HER2, all samples were unreactive (Images for p-AKT and HER2 not included).

**Table 4:** Results of immunohistochemical staining showing sample discrimination.

	chRCC		Oncocytoma		
Protein	Positive	Negative	Positive	Negative	P-value
AQP6	3/11 (28%)	8/11 (72%)	6/7 (86%)	1/7 (14%)	0.05
Parafibromin	1/11 (9%)	10/11 (91%)	5/7 (71%)	2/7 (29%)	0.01
CK7	8/11 (72%)	3/11 (27%)	1/7 (14%)	6/7 (86%)	0.05
SYNGR3	9/11 (82%)	2/11 (18%)	0/7 (0%)	7/7 (100%)	0.002
p-AKT (stromal)	5/22 (28%)	17/22 (72%)	0/8 (0%)	8/8 (100%)	0.29
p-AKT (tumor)	13/22 (59%)	8/22 (41%)	4/8 (50%)	4/8 (50%)	0.68
Extracellular HER2	0/22 (0%)	22/22 (100%)	0/8 (0%)	8/8 (100%)	NA

## Discussion

ChRCC and oncocytoma are morphologic and genetically related entities, and distinction between these two tumors is important because of their different biological behaviors. However, these entities can be difficult to distinguish morphologically. We report the derivation of a novel and useful gene predictor validated both on an internal and an independent external data-set, implying its generalizability. Our results suggest that it is possible to classify accurately histopathologically challenging tumors. The degree of accuracy achieved at 93% is reasonable for a genetic classifier. However, integration into clinical practice requires a comprehensive evaluation of these classifiers within a clinical setting, comparing clinical outcomes in routine pathologic evaluation relative to that derived from novel classifiers. This may be most practically if not most ideally done in a retrospective fashion on paraffin-embedded tissue in a large multi-institutional collaboration, which we are currently pursuing. This issue may become progressively more important with the increase in incidentally detected small tumors on radiologic surveillance, where the dilemma between observation or intervention is commonly posed.

Integrating RNA and DNA genomic data allows us to verify genomic alterations in tumor samples and distinguish the genomic signatures of different tumor subtypes. Frequent losses of chromosome 1, 2, 6, 10, 13, 17, and 21 and gains in chromosome 4, 7, 11, 12, 14q and 18q were observed in chRCC, consistent with previously reported data [18,19]. For renal oncocytoma, we show a high prevalence of chromosome 1p loss. Both chromophobe RCC and oncocytoma share this chromosomal alteration, consistent with a speculation that this may represent an early event in neoplastic transformation of a common progenitor cell.

Chromosome 1p loss represents a common cytogenetic alteration in both chRCC and renal oncocytoma identified by high-throughput SNP studies. This may suggest that this is an early event in the histogenesis of both tumors, before additional cellular events lead to malignancy in lesions that progress to chRCC, similar to chromosome 3p loss in clear cell renal cell carcinoma, which is thought to be an early event in carcinogenesis. Loss of chromosome 1p has been identified recently in renal oncocytoma [20], but this has not been previously shown to be a common cytogenetic alteration common to both entities, which is the key insight. Our delineation of the nature of chromosome 1p loss in renal oncocytoma provides the opportunity to identify novel tumor suppressor genes in future studies, and in establishing a possible carcinogenesis progression sequence.

There has been a recent advent of targeted therapies for a wide variety of cancers. Given the relative rarity of chRCC, there is no current standard of care and it is unlikely that any specific clinical trial is feasible or will be initiated. Here, we report two clinically relevant pathways--the c-erbB2/HER2 pathway and the mTOR signaling pathway--are dysregulated in chRCC on exploratory pathway analysis of mRNA expression, but our evaluation of extracellular HER2 and phospho-AKT immunohistochemical expression has not provided direct support for this mRNA finding. On a clinical trial level, in a subgroup analysis of a Phase III trial of temsirolimus, an mTOR inhibitor, in poor-prognosis RCC of all subtypes, patients of non-clear cell histology benefited as much as patients with clear cell histology, if not more [21]. Our findings do not permit a single definitive conclusion about the nature of pathway activation in these two entities. Currently, mTOR inhibitors remain a clinical standard of care for poor-risk metastatic non-clear cell renal cell carcinoma. HER2 expression has been evaluated in chromophobe RCC and oncocytoma, with distinct patterns of peptide expression varying according to epitope [22]. Interestingly, this study showed that strong intracellular HER2 expression (as defined by a 3+ expression) was strongly expressed in chromophobe RCC (9/19) but not in oncocytoma (1/11), whereas neither chromophobe RCC nor oncocytoma showed strong extracellular HER2 expression. Further evaluation of this is warranted, in conjunction with relevant fluorescent *in-situ *hybridization studies.

It has been previously reported that oxidative phosphorylation and energy pathway genes are overexpressed in chRCC and renal oncocytoma relative to the other subtypes of RCC [9]. We are able to clarify this issue, demonstrating that even between these two entities, there are major differences in quantitative expression of the same pathways discriminating the two entities. Consistent with these results, it has been recently reported that oncocytomas exhibit mitochondrial DNA mutations with clonal expansion and complex I deficiencies [23]. Oncocytoma contains a large number of mitochondria, and the overexpression of these genes involved in cellular metabolism may reflect the relative quantitative excess of the mitochondria. A similar profound modification in energy metabolism genes has been observed in thyroid oncocytomas, with high activity of the aerobic respiratory pathway [24]. It may be speculated that potential inhibition of autophagy in the chromophobe RCC may correspond to this difference as well. Rohan et al have previously reported in a smaller data-set that gene expression profiling is able to discriminate oncocytomas and chRCC [11], and has reported that vesicular transport and cell junction proteins are relatively upregulated in chRCC.

In the process of validating our high-throughput expression studies, we report three novel markers discriminating between chRCC and oncocytoma: parafibromin, aquaporin 6, and synaptogyrin 3. Parafibromin, the protein product of the *HRPT2 *tumor suppressor gene, has been reported to be downregulated in a variety of tumors [17,25], and a role has been assigned to it in the Wnt signaling pathway [26]. While the mechanism of parafibromin downregulation in parathyroid carcinoma appears to be mediated through gene mutation, this does not seem to be the mechanism in chRCC, as we have not identified any *HRPT2 *mutations after analyzing DNA samples from 5 chRCC tumors (data not shown). Similarly, other investigators have reported allelic imbalances in the HRPT2 gene in oncocytoma and chromophobe RCC, but no mutations [27]. Aquaporin 6 is an intracellular vesicle water channel protein reported to be expressed in the intercalated cells of the collecting duct [28], which is hypothesized to be the originating cell for oncocytoma and chRCC [4]. Little is known about synaptogyrin-3, a tyrosine-phosphorylated protein that is expressed in synaptic vesicles [29]. The reasons underlying the reduced expression of aquaporin 6 and increased expression of synaptogyrin-3 in chRCC, relative to oncocytoma are uncertain.

## Conclusion

In summary, we have comprehensively characterized the molecular profiles of chRCC and oncocytoma using high throughput expression and SNP profiling. We have consequently derived discriminating expression signatures, pathways, cytogenetic profiles and protein markers that are of biologic, clinical and therapeutic interest.

## Abbreviations

chRCC: Chromophobe renal cell carcinoma; SNP: single-nucleotide polymorphism; RCC: renal cell carcinoma; RMA: robust multichip average; SAM: significance analysis of microarrays; PAM: prediction analysis of microarrays; CGMA: comparative genomic microarray analysis; GO: gene ontology; CN: copy number; CNA: copy number alteration; mTOR: mammalian target of rapamycin; EGF: epidermal growth factor.

## Competing interests

The authors declare that they have no competing interests.

## Authors' contributions

MHT, HLT, JD, DM, SKK and JS performed the molecular genetic studies. MHT, HLT, KAF, EK, and DM analyzed the data. CFW, XJY, and PHT provided pathologic expertise. SG, SF, DA-O, TF, NT, MZ, GB, SD, VM, AV, and SR contributed and evaluated the samples. BTT conceived of the study and participated in its design and coordination. MHT and BTT wrote the manuscript. All authors reviewed and approved the final manuscript.

## Pre-publication history

The pre-publication history for this paper can be accessed here:

http://www.biomedcentral.com/1471-2407/10/196/prepub

## Supplementary Material

Additional file 1 **Figure S1 - Cross validated discrimination of oncocytoma and chromophobe RCC by PAM across a series of thresholds**. For derivation of a small gene classifier, we used prediction analysis of microarrays (PAM), an R implementation of nearest shrunken centroids methodology with 10-fold cross validation over 100 gene thresholds and an offset percentage of 30%. PAM yielded excellent cross-validated discrimination over a series of thresholds. Click here for file

Additional file 2 **Table S1 - Differentially expressed probe sets between oncocytoma and chromophobe RCC**. 5,210 probe sets were found to be differentially expressed between the two entities as identified using SAM at a delta of 1.4, with a false discovery rate of 0.03 corresponding to an estimated 222 probe sets. Click here for file

Additional file 3 **Table S2 - Gene ontology analyses for genes discriminating between oncocytoma and chromophobe RCC**. Gene ontology analyses highlighting that mitochondrial genes were highly overrepresented among genes relatively overexpressed in oncocytomas, whereas tight junction genes were similarly overrepresented among genes overexpressed in chRCC. Click here for file
