# Supplementary figures and images for: Genomic expression and single-nucleotide polymorphism profiling discriminates chromophobe renal cell carcinoma and oncocytoma
Source: BMC Cancer. 2010 May 12;10:196. doi: 10.1186/1471-2407-10-196 (PMC2883967; doi:10.1186/1471-2407-10-196)

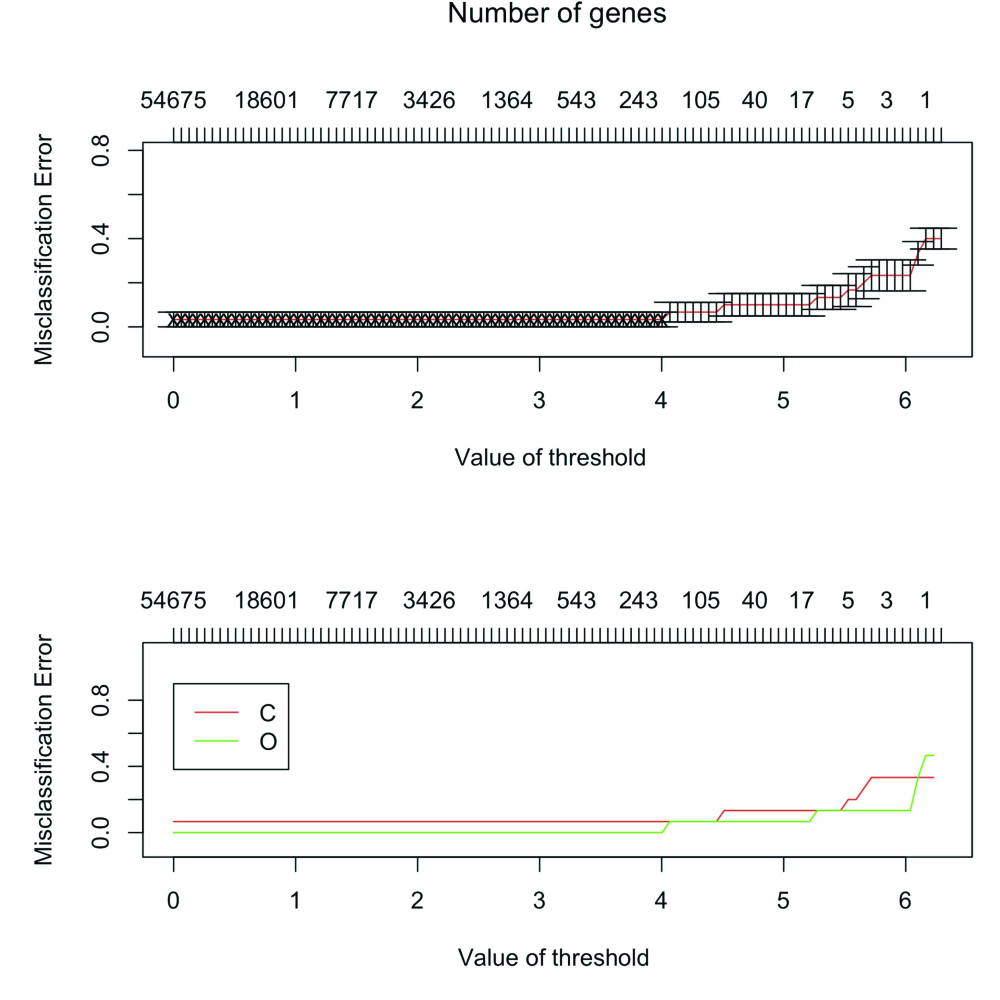

Supplement: Additional file 1 — Figure S1 - Cross validated discrimination of oncocytoma and chromophobe RCC by PAM across a series of thresholds. For derivation of a small gene classifier, we used prediction analysis of microarrays (PAM), an R implementation of nearest shrunken centroids methodology with 10-fold cross validation over 100 gene thresholds and an offset percentage of 30%. PAM yielded excellent cross-validated discrimination over a series of thresholds. [file 1471-2407-10-196-S1.TIFF]
